# Advanced Research on Glucosinolates in Food Products

**DOI:** 10.3390/foods10123148

**Published:** 2021-12-20

**Authors:** Franziska S. Hanschen, Sascha Rohn

**Affiliations:** 1 Leibniz Institute of Vegetable and Ornamental Crops (IGZ) e.V., Theodor-Echtermeyer-Weg 1, D-14979 Grossbeeren, Germany; 2 Institute of Food Technology and Food Chemistry, Technische Universität Berlin, Gustav-Meyer-Allee 25, D-13355 Berlin, Germany; rohn@tu-berlin.de

Glucosinolate-containing foods, such as vegetables from the plant order Brassicales and its derivative products, are valued for their health-beneficial properties. The latter are linked to glucosinolate hydrolysis products, such as isothiocyanates [1].

The Special Issue “Advanced Research on Glucosinolates in Food Products” aimed to collect the latest research on the impact of the whole food supply chain, including production, as well as domestic food preparation, on glucosinolates and the formation and chemistry of their breakdown products in vegetables and further foods. In this context, the consequences for human health are important, too. This Special Issue includes eleven research articles that cover research on the effect of pre-harvest factors on glucosinolates, their hydrolyzing enzymes, and the formation of volatile hydrolysis products [2,3,4,5]. Further topics include the linkage between glucosinolates and sensory aspects [2,6,7], and the effects of food preparation and follow-up reactivity [7,8,9,10]. Finally, two articles focus on the bioavailability and functional effects of isothiocyanates for human health [1,11].

Given that they are evolutionary plant defense compounds, glucosinolates and their hydrolysis in Brassicaceae vegetables are strongly affected by plant genotype and ecophysiological parameters. One of the studies described by Oloyede et al. revealed that cabbage grown in the field at lower temperatures exhibited higher myrosinase activity compared to greenhouse-grown plants, but the effect on glucosinolates and their hydrolysis products differed between cabbage morphotypes [5]. In another study, Chowdhury et al. showed that the growth conditions in a plant factory that are optimal for the plant growth of kale can differ from the optimal conditions for higher glucosinolate contents, as glucosinolate levels decrease as temperatures and humidity levels increase, though they are positively affected by increased CO_2_ levels [3]. In contrast, Bell et al. revealed a positive correlation between the presence of glucosinolates and the average growth temperature of rocket (*Diplotaxis tenuifolia* and *Eruca sativa*), demonstrating this with multiple leaf cuts, showing how the process affected the perception of pepperiness, bitterness, and hotness, which in turn reduced consumer acceptance [2]. These findings pose a challenge for the aim to increase health-promoting isothiocyanate levels, as this could reduce consumer acceptance and thereby the consumption of these products. Growth conditions also seem to affect glucosinolate hydrolysis outcomes, as in a study with commercial white and red cabbages purchased over a 3-month period. Red cabbages especially showed higher isothiocyanate release in late summer compared to cabbages purchased in late autumn, with the latter also being more intense producers of nitriles and epithionitriles [4].

The processing of these vegetables during food preparation can considerably affect glucosinolates, their product formation, and also the flavor of the products. Thermal treatments inactivate myrosinase, thereby stopping enzymatic glucosinolate hydrolysis, resulting in diminished isothiocyanate formation [10]. Stir-frying maintains myrosinase activity and, consequently, reduced glucosinolate contents were observed in stir-fried foods. In contrast, steaming retains glucosinolate levels the best and often favors a forced isothiocyanate formation [10]. However, a high content of glucosinolates in boiled vegetables can affect consumer acceptance as well, because intact glucosinolates were positively correlated with a bitter taste [7]. In contrast, isothiocyanates are very reactive compounds and undergo a transformation with nucleophiles, thereby reducing their contents in a food matrix and forming reaction products. Krell et al. investigated the fate of glucosinolates during the baking of bread enriched with nasturtium (*Tropaeolum majus* L.). Next to the thermal degradation of glucosinolates to nitriles, Krell et al. showed that isothiocyanates react with the lysine residues of the wheat proteins in the bread crumb [9]. Another example of follow-up reactivity is the isothiocyanate 4-methylthio-3-butenyl isothiocyanate being known as a precursor to the yellow pigment 2-[3-(2-thioxopyrrolidin-3-ylidene)methyl]-tryptophan, present in the traditional Japanese salted radish root ‘takuan-zuke’. Kobayashi et al. studied the formation mechanism and found that the yellowing of takuan-zuke is accelerated at pH values below 5 and that the color of air-dried takuan-zuke was deeper than that of the salt-pressed product. Moreover, the findings in the study described by Kobayashi et al. led to the assumption that a so far unknown pigment is responsible for the color, as 2-[3-(2-thioxopyrrolidin-3-ylidene)methyl]-tryptophan levels were too low to account for the color alone [8].

When eating Brassicales plants or products thereof, glucosinolates, and especially their isothiocyanates, are valued for their positive effects on human health. However, conversion to isothiocyanates and bioavailability determines the effects of these compounds tremendously. Shekarri and Dekker described a physiologically based model to simulate the bioavailability and absorption kinetics of sulforaphane (4-(methylsulfinyl)butyl isothiocyanate, which is found in broccoli and also in red cabbage). This model represents a preliminary step in enabling the prediction of the biological effect of isothiocyanates and can be used in the growing field of personalized nutrition [11]. Novel insights into the immunomodulating effects of watercress (*Nasturtium officinale*) isothiocyanates on exercise-induced inflammation are presented in a human intervention study described by Schulze et al. [1] After the consumption of fresh watercress (a source of 2-phenylethyl isothiocyanate), a mild pro-inflammatory reaction was observed, though the immune response was more pronounced for both pro-inflammatory and anti-inflammatory markers after an exercise unit compared to a control meal. However, during the recovery phase, watercress consumption led to a stronger anti-inflammatory downregulation of the pro-inflammatory cytokines IL-6 and TNF-α. Thus, the study came to the conclusion that fresh watercress causes a stronger pro-inflammatory response and anti-inflammatory counter-regulation during and after exercise [1].

Due to the inclusion of well-designed studies and well-written articles, this Special Issue offers a valuable contribution to the ambition of producing vegetables rich in valuable glucosinolates as a source of health-promoting isothiocyanates. However, the research also reveals that this ambition might negatively affect consumer liking and acceptance, and therefore this needs to be considered in the future as well. New insights into the effect of food processing on glucosinolates and the formation and fate of their breakdown in follow-up products are presented. These processes are still far away from being fully understood. Evaluating the role of glucosinolates in supporting human health still remains challenging.
